# Randomized phase 3 efficacy and safety trial of proposed pegfilgrastim biosimilar MYL-1401H in the prophylactic treatment of chemotherapy-induced neutropenia

**DOI:** 10.1007/s00277-019-03639-5

**Published:** 2019-03-01

**Authors:** Cornelius F. Waller, Gopinath M. Ranganna, Eduardo J. Pennella, Christopher Blakeley, Miguel H. Bronchud, Leonard A. Mattano Jr, Oleksandr Berzoy, Nataliia Voitko, Yaroslav Shparyk, Iryna Lytvyn, Andriy Rusyn, Vasil Popov, István Láng, Katrin Beckmann, Rajiv Sharma, Mark Baczkowski, Mudgal Kothekar, Abhijit Barve

**Affiliations:** 1 Department of Haematology, Oncology and Stem Cell Transplantation, University Medical Centre Freiburg and Faculty of Medicine, University of Freiburg, Hugstetter Street 55, 79106 Freiburg, Germany; 2 Global Clinical Research & Development, Mylan, Prestige Tech Park Platina-3, 7th to 12th Floor, Kadubesanahalli, Bangalore, 560103 India; 3 Global Clinical Research, Mylan, 1000 Mylan Boulevard, Canonsburg, PA 15317 USA; 4 Medical and Scientific Affairs, Worldwide Clinical Trials, 172 Tottenham Court Road, Fitzrovia, London, W1T 7DL UK; 5 GenesisCare Corachan Institute of Oncology, Buïgas 19, 08017 Barcelona, Spain; 6 HARP Pharma Consulting, LLC, 184 Masons Island Road, Mystic, CT 06355 USA; 7 Mammalogy Center, Odessa Regional Hospital, Akademika Zabolotnogo Str. 26, Odessa, 65025 Ukraine; 8 Chemotherapy II, Kyiv City Clinical Oncological Centre, Kyiv, Street, Verkhovyna, 69, Kiev, 03115 Ukraine; 9 Department of Chemotherapy, Lviv State Regional Treatment and Diagnostics Oncology Center, Lviv Street, Pekarskaya, 69, Lviv, 79010 Ukraine; 10 Department of Chemotherapy, Dnipropetrovsk Regional Clinical Oncology Center, Kosmicheskaja Street, 21, Dnepropetrovsk, 49100 Ukraine; 11 Department of Chemotherapy, Transkarpathian Regional University Oncology Clinic, Narodna Square, 3, Uzhgorod, 88000 Ukraine; 12 Department of Medical Oncology and Palliative Care, SHATOD Dr. Marko Аntonov Markov, Varna EOOD, SHOP “Tsar Osvoboditel” Boulevard 100000, 9000 Varna, Bulgaria; 13 Department of Medical Oncology and Clinical Pharmacology B, National Institute of Oncology Országos Onkológiai Intézet, Budapest Ráth György u. 7-9, Budapest, 1122 Hungary; 14 Global Clinical Operations, Mylan Healthcare GmbH, Freundallee 9A, 30173 Hannover, Germany; 15 Global Product Safety and Risk Management, Mylan, Building 4 Trident Place, Mosquito Way, Hatfield, AL10 9UL UK; 16 Product Safety and Risk Management, Mylan, 781 Chestnut Ridge Road, Morgantown, WV 26505 USA; 17 Clinical Development, Biocon Research Ltd, 131, Jigani Road, RK Twp, Bommasandra Industrial Area, Bangalore, Karnataka 560099 India

**Keywords:** Pegfilgrastim, Biosimilar, Febrile neutropenia, Chemotherapy-induced neutropenia

## Abstract

Pegfilgrastim is indicated for reducing the duration of neutropenia and incidence of febrile neutropenia in patients receiving cytotoxic chemotherapy. Here, safety and efficacy of MYL-1401H, a proposed pegfilgrastim biosimilar, were investigated as prophylaxis for chemotherapy-induced neutropenia. This was a phase 3, multicenter, randomized, double-blind, parallel-group equivalence trial of MYL-1401H vs European Union–sourced reference pegfilgrastim. Patients with newly diagnosed stage II/III breast cancer eligible to receive (neo) adjuvant chemotherapy with docetaxel/doxorubicin/cyclophosphamide every 3 weeks for 6 cycles were enrolled and randomized 2:1 to 6 mg of MYL-1401H or reference pegfilgrastim 24 h (+ 2-h window after the first 24 h) after the end of chemotherapy. The primary efficacy endpoint was the duration of severe neutropenia in cycle 1 (i.e., days with absolute neutrophil count (ANC) < 0.5 × 10^9^/L). Mean (standard deviation (SD)) duration of severe neutropenia in MYL-1401H and reference pegfilgrastim groups was 1.2 days (0.93) and 1.2 days (1.10), respectively. The 95% CI for least squares mean difference (− 0.285, 0.298) was within the predefined equivalence range of ± 1 day. Secondary endpoints, including grade ≥ 3 neutropenia (frequency, 91% and 82% for MYL-1401H and reference pegfilgrastim, respectively), time to ANC nadir (mean (SD), 6.2 (0.98) and 6.3 (1.57) days), and duration of post-nadir recovery (mean (SD), 1.9 (0.85) and 1.7 (0.91) days) were comparable. Overall safety profiles of the study drugs were comparable. MYL-1401H demonstrated equivalent efficacy and similar safety to reference pegfilgrastim and may be an equivalent option for reducing incidence of neutropenia. (ClinicalTrials.gov, NCT02467868; EudraCT, 2014-002324-27).

**Electronic supplementary material:**

The online version of this article (10.1007/s00277-019-03639-5) contains supplementary material, which is available to authorized users.

## Introduction

Biologics have great therapeutic promise but are limited by cost and availability [1]. As biologics lose patent protection, biosimilar development may reduce costs and expand access [1–3]. Recently, the European Medicines Agency (EMA) and the US Food and Drug Administration (FDA) established guidance for biosimilar development and approval [4, 5]. Preclinical characterization, pharmacokinetic/pharmacodynamic (PK/PD), safety, and efficacy studies should be conducted to demonstrate equivalence to reference product [4, 5].

Filgrastim, which has been used for febrile neutropenia (FN) prophylaxis in patients receiving myelosuppressive chemotherapy for > 25 years, was among the first drugs to have biosimilar versions approved by the EMA (2008) and FDA (2015) [6–8]. Filgrastim for chemotherapy-induced neutropenia (CIN) prophylaxis can reduce infection-related mortality in patients with cancer and minimize chemotherapy dose reductions and delays [9, 10]. Clinical studies have shown that filgrastim biosimilars have pharmacologic, safety, and efficacy profiles comparable to the originator product [11–15]. Accordingly, guidelines now indicate filgrastim biosimilars are appropriate for FN prophylaxis in patients receiving myelosuppressive chemotherapy [15, 16].

While filgrastim is administered daily, the pegylated form, pegfilgrastim, is only administered once per chemotherapy cycle [16, 17]. Pegfilgrastim was approved in 2002 for CIN prophylaxis, and significant research has gone into pegfilgrastim biosimilar development [3]. Recent preclinical and PK/PD data have supported biosimilarity between originator pegfilgrastim (Neulasta®; Amgen Inc., Thousand Oaks, CA) and the proposed biosimilar MYL-1401H [18]. Here, we present data from a phase 3 efficacy and safety trial conducted to confirm equivalence of MYL-1401H with European Union (EU)–sourced reference pegfilgrastim for CIN prophylaxis.

## Materials and methods

### Study design

This phase 3, randomized, double-blind, parallel-group trial evaluated equivalence of MYL-1401H and reference pegfilgrastim (Neulasta) in patients with breast cancer eligible to receive neoadjuvant or adjuvant TAC (docetaxel 75 mg/m^2^, doxorubicin 50 mg/m^2^, cyclophosphamide 500 mg/m^2^) chemotherapy (ClinicalTrials.gov, NCT02467868; EudraCT, 2014-002324-27; Supplementary Fig. 1 in Online Resource). Patients were screened within 4 weeks of chemotherapy initiation, and eligible patients were randomized 2:1 to MYL-1401H or reference pegfilgrastim via interactive voice/web response system and stratified by age and country. Reference pegfilgrastim (EMA approved) was obtained from European sources.

Randomized patients underwent 6 planned chemotherapy cycles every 3 weeks. In each cycle, chemotherapy was administered on day 1 and a single subcutaneous 6-mg dose of MYL-1401H or reference pegfilgrastim was administered 24 h (+ 2-h window after the first 24 h) after the end of chemotherapy. Only the pharmacist preparing the doses and the person (not the principal investigator) administering treatment were unblinded.

All patients were aged ≥ 18 years with newly diagnosed stage II/III breast cancer and adequate staging workup and surgery if receiving adjuvant therapy. Patients were required to be chemotherapy and radiotherapy naive with Eastern Cooperative Oncology Group performance status ≤ 1 and absolute neutrophil count (ANC) ≥ 1.5 × 10^9^/L at baseline. Patients previously exposed to filgrastim products were excluded as they can potentially develop antidrug antibodies (ADA) that may interfere with study assessments [14].

The choices of patient population and chemotherapy regimen were determined using guidance from regulatory agencies. As recommendations support routine use of filgrastim in patients receiving chemotherapy regimens that are associated with a > 20% risk of FN, prophylactic treatment with pegfilgrastim in this patient population is consistent with current guidelines [15, 16].

The clinical study protocol, informed consent forms, and all other appropriate study-related documents were approved by local independent ethics committees and institutional review boards, as applicable. The study was conducted in accordance with International Council for Harmonization Guideline for Good Clinical Practice and the Declaration of Helsinki at 25 sites in Bulgaria, Georgia, Hungary, and Ukraine between March 25, 2015, and February 9, 2016.

### Endpoints

The primary efficacy endpoint was the duration of severe neutropenia (DSN) in cycle 1, defined as days with ANC < 0.5 × 10^9^/L. During cycle 1, ANC was determined from blood samples collected daily for 15 days after chemotherapy administration. In subsequent cycles, ANC was measured on days 8, 11, and 15.

Measurements of ANC were used to calculate secondary efficacy endpoints: frequency of grade 3 or 4 neutropenia, depth and time to ANC nadir and rate of recovery, and rate of FN. Febrile neutropenia was defined per European Society for Medical Oncology as ANC < 0.5 × 10^9^/L or expected to fall below 0.5 × 10^9^/L, with a single oral temperature > 38.5 °C or 2 consecutive readings >38.0 °C for 2 h. Delay, reduction, and omission of chemotherapy doses were also recorded.

All adverse events (AEs), regardless of relationship to study drug, were recorded through 28 days after the last administration of MYL-1401H or reference pegfilgrastim. Adverse events were graded using the National Cancer Institute Common Terminology Criteria for Adverse Events (version 4.03). In the first 2 cycles, bone pain was measured daily for the first 15 days by the Brief Pain Inventory (BPI) short form [19]. Injection site reactions were documented during all cycles. Blood samples for assessment of ADA and neutralizing antibodies (NAb) were collected on day 1, cycle 1, and at the end of cycles 2, 4, and 6.

### Statistical analysis

A sample size of 135 patients randomized 2:1 (90 and 45 patients treated with MYL-1401H and reference pegfilgrastim, respectively) was required to provide 90% power to declare equivalence between MYL-1401H and reference pegfilgrastim for analysis of DSN in cycle 1. This sample size assumed that the mean DSN was 1.7 days in cycle 1 for both MYL-1401H and reference pegfilgrastim, with a common standard deviation (SD) of 1.5 days [20, 21], and the difference between mean DSN was analyzed with a 2-sided 95% CI. The required sample size of 135 patients was increased to 189 to allow for attrition and ensure that safety was assessed in ~100 patients receiving MYL-1401H for 6 cycles per EMA requirement.

Primary efficacy analysis was conducted in the per protocol (PP) population (randomized patients without major protocol deviations who received ≥ 1 study drug dose). Analysis of variance (ANOVA) model with treatment as an independent factor was used to produce a 2-sided 95% CI for difference in the least square mean DSN. Equivalence was declared if the CI was completely within the range of ± 1 day. The equivalence margin was in alignment with guidelines provided by regulatory authorities (FDA and EMA) [22]. Secondary efficacy endpoints were analyzed descriptively. Chi-square test was used to assess the difference in the occurrence of FN between treatment groups. Safety was analyzed in patients who received ≥ 1 study drug dose (safety population). The intention-to-treat (ITT) population included all randomized patients. All statistical analyses were performed using SAS® (Cary, NC).

## Results

### Patient disposition

Of 207 patients screened, 194 were randomized, 127 received MYL-1401H, and 67 received reference pegfilgrastim (Supplementary Fig. 2 in Online Resource). All randomized patients completed cycle 1 and were included in both the ITT and safety populations. One patient in the MYL-1401H group took a prohibited concomitant medication and was excluded from the PP population. All 6 chemotherapy cycles were completed by 120 patients in the MYL-1401H group and 66 in the reference pegfilgrastim group.

### Baseline characteristics

No differences were observed in baseline characteristics between groups (Table 1). Median age (range) was 49 years (25–79) in the MYL-1401H group and 50 years (29–68) in the reference pegfilgrastim group. All 67 (100%) patients in the reference pegfilgrastim group and 126 (99%) in the MYL-1401H group were female. All 194 patients were white.

**Table 1:** Patient Characteristics (ITT Population)

Parameter	MYL-1401H (*N* = 127)	Reference (*N* = 67)	Overall (*N* = 194)
Age, *y*			
Mean (SD)	50 (11)	50 (10)	50 (10)
Median (range)	49 (25–79)	50 (29–68)	50 (25–79)
Age group, y, *n* (%)			
< 50	64 (50)	32 (48)	96 (50)
50–65	56 (44)	30 (45)	86 (44)
> 65	7 (6)	5 (8)	12 (6)
White, *n* (%)	127 (100)	67 (100)	194 (100)
Sex, *n* (%)			
Female	126 (99)	67 (100)	193 (99)
Male	1 (1)	0 (0)	1 (1)
TNM stage at diagnosis, *n* (%)			
IIa	34 (27)	15 (22)	49 (25)
IIb	42 (33)	22 (33)	64 (33)
IIIa	26 (21)	16 (24)	42 (22)
IIIb	6 (5)	7 (10)	13 (7)
IIIc	19 (15)	7 (10)	26 (13)
Indicated treatment, *n* (%)			
Adjuvant	73 (58)	43 (64)	116 (60)
Neoadjuvant	54 (42)	24 (36)	78 (40)

*ITT* intention-to-treat, *SD* standard deviation, *TNM* tumor node metastasis staging system

### Efficacy

#### Primary endpoint

In the PP population, mean (SD) DSN for MYL-1401H was 1.2 days (0.93), and median (range) DSN was 1.0 day (0–5; Table 2). Mean (SD) DSN for reference pegfilgrastim was 1.2 days (1.10), and median (range) DSN was 1.0 day (0–4). The 95% CI (− 0.285, 0.298) for the difference in LS mean DSN of MYL-1401H and reference pegfilgrastim was entirely within the prespecified equivalence range of ± 1 day based on the ANOVA model. Therefore, equivalent efficacy of MYL-1401H and reference pegfilgrastim was concluded.

**Table 2:** Efficacy Endpoints

Frequency, depth, and time of neutropenia in cycle 1 (PP population)	MYL-1401H (*N* = 126)	Reference (*N* = 67)
DSN, mean (SD), days	1.2 (0.9)	1.2 (1.1)
DSN, LS mean (SE), days	1.3 (0.1)	1.3 (0.2)
Grade 3 neutropenia, *n* (%)^a,b^	20 (15.9)	12 (17.9)
Grade 4 neutropenia, *n* (%)^b,c^	94 (74.6)	43 (64.2)
ANC nadir, mean (SD), 10^9^/L	0.40 (0.5)	0.78 (1.4)
ANC nadir, median (range), 10^9^/L	0.21 (0.0–2.5)	0.27 (0.0–6.7)
Duration of post-nadir ANC recovery within ≤ 3 days, *n* (%)	121 (97)^d^	67 (100)
Rate of febrile neutropenia in cycle 1 (ITT population)	MYL-1401H (*N* = 127)	Reference (*N* = 67)
Rate of febrile neutropenia, n (%)	5 (4)	1 (2)
Frequency of neutropenia across all cycles (ITT population)	MYL-1401H (*N* = 127)	Reference (*N* = 67)
Grade 3 neutropenia, *n* (%)^a,b^	17 (13)	7 (10)
Grade 4 neutropenia, *n* (%)^b,c^	103 (81)	49 (73)
Febrile neutropenia, *n* (%)^e^	7 (6)	1 (2)
Frequency of chemotherapy doses reduced, omitted, or delayed across all cycles (ITT population)	MYL-1401H (*N* = 127)	Reference (*N* = 67)
Related to neutropenia, febrile neutropenia, or documented infections, *n* (%)	5 (4)	1 (2)

*ANC* absolute neutrophil count, *DSN* duration of severe neutropenia, *ESMO* European Society for Medical Oncology, *ITT* intention-to-treat, *LS* least squares, *PP* per protocol, *SD* standard deviation, *SE* standard error

^a^ANC < 1.0 × 10^9^/L.

^b^Only the highest grade neutropenia experienced was reported

^c^ANC < 0.5 × 10^9^/L.

^d^Percentage calculated using the 125 patients with a confirmed post-nadir ANC recovery

^e^Out of 8 patients with febrile neutropenia, 3 met the ESMO definition, 1 patient did not, and 4 other patients had insufficient data to confirm febrile neutropenia, but all patients were considered to have febrile neutropenia for the data analysis

#### Secondary endpoints

**Fig. 1:**
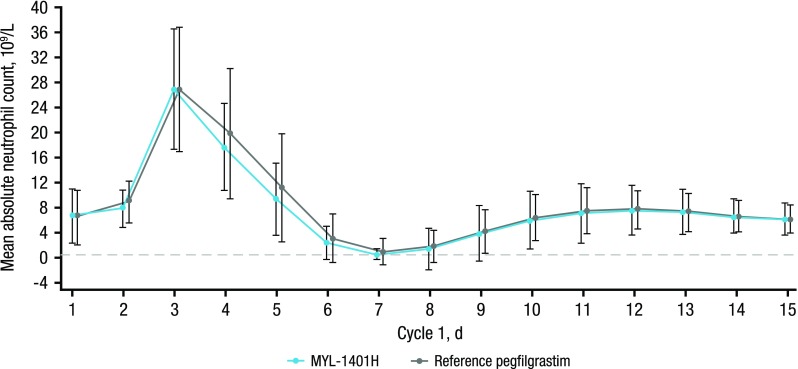
Mean ANC over time by treatment in cycle 1 (PP population). *ANC* absolute neutrophil count, *PP* per protocol

Mean ANC profiles of MYL-1401H– and reference pegfilgrastim–treated patients were similar in cycle 1 (PP population; Fig. 1). Mean (SD) time to ANC nadir was 6.2 days (0.98) and 6.3 days (1.57) for MYL-1401H and reference pegfilgrastim, respectively. Median (range) ANC nadir was 0.21 × 10^9^/L (0.0–2.5 × 10^9^/L) and 0.27 × 10^9^/L (0.0–6.7 × 10^9^/L) for MYL-1401H and reference pegfilgrastim, respectively. All patients in cycle 1 receiving reference pegfilgrastim had post-nadir ANC recovery. Post-nadir ANC recovery could not be established in 1 patient (1%) receiving MYL-1401H because the patient did not have 2 recovery observations before the end of cycle 1. However, all evaluable patients (i.e., those with 2 recovery observations) receiving MYL-1401H had post-nadir ANC recovery. In patients with confirmed post-nadir ANC recovery, mean (SD) time to recovery was 1.9 days (0.85) and 1.7 days (0.91) for MYL-1401H and reference pegfilgrastim, respectively. Overall, 97% of patients (121/125) receiving MYL-1401H and all patients receiving reference pegfilgrastim with a confirmed post-nadir ANC recovery in cycle 1 had recovery time of ≤ 3 days.

In cycle 1, grade ≥ 3 neutropenia occurred in 91% of patients (114/126) receiving MYL-1401H and 82% (55/67) receiving reference pegfilgrastim; grade 4 neutropenia occurred in 75% of patients (94/126) receiving MYL-1401H and 64% (43/67) receiving reference pegfilgrastim (PP population). However, in a majority of patients, grade 4 neutropenia did not recur in all cycles. Overall, in 19% of patients receiving MYL-1401H and 27% receiving reference pegfilgrastim, no grade 4 neutropenia occurred. Sixty-one patients (48%) receiving MYL-1401H and 31 (46%) receiving reference pegfilgrastim had grade 4 neutropenia in 1 or 2 cycles only. Less than 10% of patients had grade 4 neutropenia in all 6 cycles: 7% and 9% receiving MYL-1401H and reference pegfilgrastim, respectively. No prolonged neutropenia (> 10 days) was reported, and profound neutropenia (< 0.1 × 10^9^/L) incidence was similar between groups. Across all cycles, FN occurred in 6% of patients (7/127) receiving MYL-1401H and 2% (1/67) receiving reference pegfilgrastim (ITT population). Most FN events occurred during cycle 1: MYL-1401H, 4% (5/127) and reference pegfilgrastim, 2% (1/67). Although there were numerical differences in FN rates, these differences were not significant for cycle 1 (difference, 2.4; 95% CI, − 2.0, 6.9; *P* = 0.35) or 6 cycles (difference, 4.0; 95% CI, − 0.9, 8.9; *P* = 0.18). Post hoc analysis showed that MYL-1401H is noninferior to reference pegfilgrastim for FN incidence, assuming a noninferiority margin of 9%. All FN incidences were of short duration and resolved within 5 days of onset; no infections required treatment or rescue therapy with filgrastim, and no infection-related mortality was observed. Reductions, omissions, or delays in chemotherapy related to neutropenia, FN, or documented infections occurred in 4% of patients (5/127) receiving MYL-1401H and 2% (1/67) receiving reference pegfilgrastim (ITT population).

The isolated differences between groups in incidence of grade ≥ 3 neutropenia; occurrence of FN; and reductions, omissions, or delays in chemotherapy were observed in exploratory analyses not powered for comparison. No significant infections or sepsis were reported in either group during FN or grade ≥ 3 neutropenia events.

### Safety

Similar rates of treatment-emergent AEs (TEAEs) were observed between groups (Table 3). A total of 806 TEAEs were reported in 114 (90%) patients receiving MYL-1401H, and 414 TEAEs were reported in 58 (87%) patients receiving reference pegfilgrastim. One patient receiving MYL-1401H experienced a grade ≥ 4 TEAE (grade 4 FN), which was considered unrelated to study drug. No patients receiving reference pegfilgrastim experienced any grade ≥ 4 TEAEs. No MYL-1401H–treated patients reported an injection site reaction. One (2%) patient receiving reference pegfilgrastim had injection site redness on day 2, cycle 2, and 1 (2%) had bruising on day 8, cycle 2 and day 8, cycle 3. No deaths, treatment-related discontinuations, or suspected unexpected serious adverse reactions occurred in either group.

**Table 3:** TEAEs by Preferred Term Across All Cycles (Safety Population)

	MYL-1401H (*N* = 127)	Reference (*N* = 67)
TEAEs, *n*	806	414
Patients with TEAEs, *n* (%)	114 (90)	58 (87)
Patients with TEAEs grade ≥ 4, *n* (%)	1 (1)^a^	0 (0)
TEAEs occurring in ≥ 5% patients in either treatment group, *n* (%)		
Alopecia	76 (60)	36 (54)
Bone pain	51 (40)	24 (36)
Nausea	37 (29)	25 (37)
Asthenia	23 (18)	10 (15)
Fatigue	19 (15)	16 (24)
Diarrhea	16 (13)	12 (18)
Thrombocytopenia	14 (11)	6 (9)
Anemia	14 (11)	9 (13)
Vomiting	12 (9)	7 (10)
Headache	12 (9)	8 (12)
Stomatitis	11 (9)	2 (3)
Thrombocytosis	8 (6)	0 (0)
Decreased appetite	8 (6)	0 (0)
Febrile neutropenia	7 (6)	1 (1)
Alanine aminotransferase increased	7 (6)	8 (12)
Aspartate aminotransferase increased	7 (6)	7 (10)
Platelet count decreased	7 (6)	5 (7)
Pyrexia	3 (2)	5 (7)
Abdominal pain	3 (2)	4 (6)

TEAE, treatment-emergent adverse event

^a^Grade 4 febrile neutropenia

For both groups, mean highest pain score, assessed by averaging individual patient responses to question 3 (i.e., worst pain in the last 24 h) of the BPI in cycle 1 (Supplementary Fig. 3 in Online Resource), was reported on day 4, followed by plateauing and eventual decline by day 15. Bone pain reported by patients was similar between groups, and no patients discontinued because of bone pain. However, naproxen use for bone pain management in cycle 1 was higher with reference pegfilgrastim (19 (28%)) than MYL-1401H (25 (20%)).

Of the patients in the safety population with available baseline immunogenicity results, 15% (19/126) receiving MYL-1401H and 18% (12/67) receiving reference pegfilgrastim were ADA positive before dosing. Of those with available immunogenicity results after treatment initiation, 1% (1/125) receiving MYL-1401H and 3% (2/67) receiving reference pegfilgrastim were ADA positive. Most ADA-positive samples before treatment were against the polyethylene glycol moiety. No ADA-positive patients were Nab positive. Only 1 (2%) ADA-positive result for reference pegfilgrastim was due to seroconversion, defined as ADA negative at baseline but with subsequent positive response. The MYL-1401H group had no treatment-induced ADA-positive patients.

## Discussion

The study met the primary endpoint as MYL-1401H efficacy was equivalent for reference pegfilgrastim. The DSN was equivalent for MYL-1401H and reference pegfilgrastim, and mean DSN in cycle 1 was similar to other studies of both biosimilar and reference pegfilgrastim [20, 21, 23]. Profiles for ANC were similar between groups throughout all chemotherapy cycles. Robust and correlative PK/PD studies are sensitive at detecting subtle differences in biosimilar efficacy. Previous PK/PD analysis of MYL-1401H demonstrated equivalence with reference pegfilgrastim [18], consistent with efficacy data from this study.

A slightly higher incidence of grade 4 neutropenia occurred with MYL-1401H (75%) vs reference pegfilgrastim (64%). However, grade 4 neutropenia recurred across all cycles in only 7% and 9% of patients receiving MYL-1401H and reference pegfilgrastim, respectively. The majority of grade 4 neutropenia events lasted < 2 days in both groups. The incidence of FN was slightly higher in the MYL-1401H treatment group; however, the difference was not statistically significant. There were no documented infections associated with grade 4 FN, and few patients (MYL-1401H, 5 (4%); reference pegfilgrastim, 1 (2%)) had their chemotherapy dose modified. Historical data for pegfilgrastim from other studies with similar populations and chemotherapy regimens have shown that incidence of FN with reference pegfilgrastim was up to 10% in cycle 1 and 13% in all cycles, with a low mean depth of ANC nadir [23, 24], similar to the range seen in patients receiving MYL-1401H. For example, mean depth of ANC nadir was 0.49 × 10^9^/L (SD, 0.72; median, 0.24; range, 0.0–4.4) and 0.44 × 10^9^/L (SD, 0.57; median, 0.30; range, 0.0–3.8) in patients receiving LA-EP2006 or reference pegfilgrastim, respectively [23]. Therefore, isolated differences in grade 4 neutropenia, FN, and ANC nadir between treatment groups were not considered clinically significant and possibly due to sample size limitations.

Both products were generally well tolerated, and only minor differences in TEAE rates and secondary efficacy endpoints were observed, which were not clinically meaningful. No unexpected serious adverse reactions occurred. One of the most frequently reported treatment-related TEAEs was bone pain, and incidence of bone pain was similar to that reported in other studies of pegfilgrastim in patients receiving myelosuppressive chemotherapy other than TAC (range, 25–38%) [25]. Rare, serious side effects of G-CSF therapy [26], such as splenomegaly, acute respiratory distress syndrome, capillary leak syndrome, and severe allergic reactions, were not observed in this study. There are limitations when comparing these results with those from similar studies because of varying patient populations, chemotherapy regimens, and other factors related to study design. On balance, the efficacy and safety results from this study are in accordance with published literature on biosimilar G-CSF products [11–14, 18, 23, 24].

## Conclusions

This study demonstrated that MYL-1401H is equivalent in efficacy to originator pegfilgrastim for prophylaxis of CIN in patients with breast cancer treated with neoadjuvant or adjuvant TAC chemotherapy, with no clinically meaningful differences in safety. Given the equivalence between groups regarding primary and secondary endpoints and known clinical benefits of G-CSF, these results can be applied to many chemotherapy regimens that result in high rates of FN, independent of tumor type.

## Electronic supplementary material

ESM 1 (PDF 174 kb)

## Data Availability

Anonymized individual participant data and study documents can be requested for further research from Joseph Capasso (Joseph.Capasso@mylan.com).
