# Supplementary material for: Randomized phase 3 efficacy and safety trial of proposed pegfilgrastim biosimilar MYL-1401H in the prophylactic treatment of chemotherapy-induced neutropenia
Source: Ann Hematol. 2019 Mar 1;98(5):1217–24. doi: 10.1007/s00277-019-03639-5 (PMC6469669; doi:10.1007/s00277-019-03639-5)

ELECTRONIC SUPPLEMENTARY MATERIAL

Randomized Phase 3 Efficacy and Safety Trial of Proposed Pegfilgrastim Biosimilar MYL-1401H in the Prophylactic Treatment of Chemotherapy-Induced Neutropenia

Annals of Hematology

Cornelius F. Waller, Gopinath M. Ranganna, Eduardo J. Pennella, Christopher Blakeley, Miguel H. Bronchud, Leonard A. Mattano Jr, Oleksandr Berzoy, Nataliia Voitko, Yaroslav Shparyk, Iryna Lytvyn, Andriy Rusyn, Vasil Popov, István Láng, Katrin Beckmann, Rajiv Sharma, Mark Baczkowski, Mudgal Kothekar, Abhijit Barve

Prof. Dr. Cornelius F. Waller
Department of Haematology, Oncology and Stem Cell Transplantation
University Medical Centre Freiburg and Faculty of Medicine
University of Freiburg
Freiburg, Germany
cornelius.waller@uniklinik-freiburg.de

SUPPLEMENTARY FIGURES

Supplementary Fig. 1 Study design. During the 18-week treatment period, patients were administered chemotherapy (star) every 3 weeks. Pegfilgrastim (arrow) was administered 24 hours after chemotherapy. DSN, duration of severe neutropenia

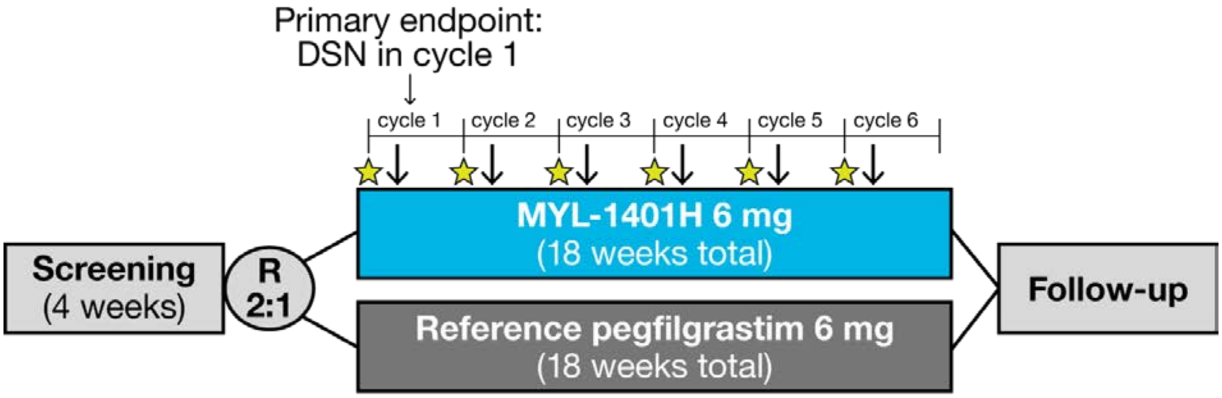

Supplementary Fig. 2 Patient disposition

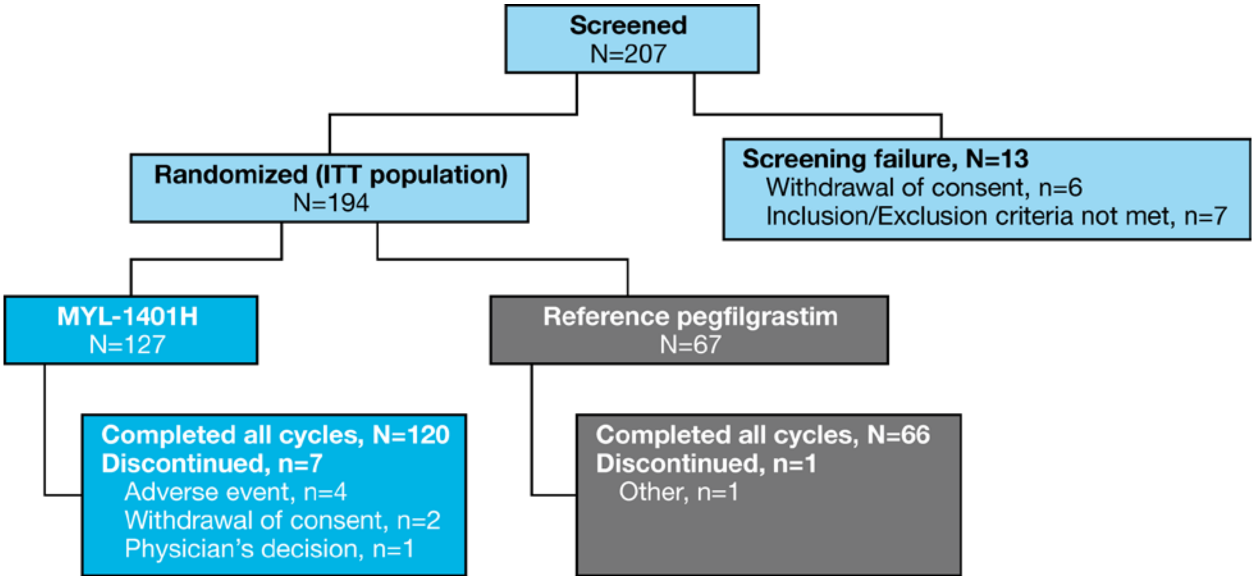

Supplementary Fig. 3 Worst reported pain in the last 24 hours by day during cycle 1 as determined by patients' responses to Brief Pain Inventory question 3 (safety population). Pain is reported on a scale of 0 (no pain) to 10 (pain as bad as you can imagine). SD, standard deviation

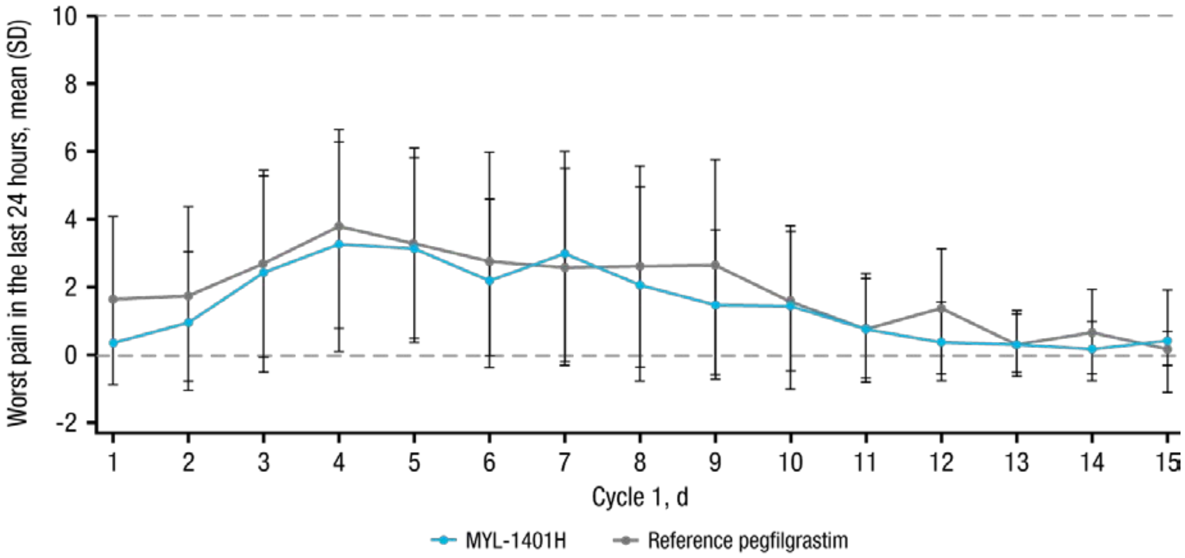

Supplement: Supplementary file 1 — (PDF 174 kb) [file 277_2019_3639_MOESM1_ESM.pdf]
